# Biomarkers in the evaluation of cardiac involvement in systemic sclerosis

**DOI:** 10.1515/rir-2024-0013

**Published:** 2024-07-15

**Authors:** Mohamad Fadhli Bin Masri, Sue-Ann Ng, Calvin WL Chin, Andrea HL Low

**Affiliations:** Department of Rheumatology and Immunology, Singapore General Hospital, Bukit Merah, Singapore; SingHealth Duke-NUS Medicine Academic Clinical Programme, Duke-NUS Medical School, Singapore, Singapore; Department of Cardiology, National Heart Centre Singapore, Singapore, Singapore

**Keywords:** systemic sclerosis, biomarkers, cardiac

## Abstract

Systemic sclerosis is a multisystemic disease for which the heart can be affected leading to cardiac complications and mortality. Up to 80% of patients with systemic sclerosis have cardiac involvement with varying levels of severity. Several molecules have been identified that can be used as markers of cardiac involvement. These biomarkers can arise directly from the heart due to cardiac damage from the disease such as cardiac troponins or from the underlying dysregulated immune process itself such as the proinflammatory cytokines including interleukin (IL)-6. This review aims to summarize the evidence on currently known biomarkers that are can be diagnostic, prognostic or predictive of primary cardiac involvement in systemic sclerosis. We also highlight potential new biomarkers based on the current understanding of the disease process. Clinical use of these markers can benefit patients through earlier identification of those with cardiac involvement, many of whom can be asymptomatic in the early stage, with higher risk of complications, with the overall goal to improve outcomes of these affected patients.

## Introduction

Systemic sclerosis (SSc), is a multisystemic disease characterized by immune dysregulation, vasculopathy and fibrosis. The disease process is mediated through a continuous cycle of chronic hypoxia and inflammation resulting in damage to the vasculature and continued fibrosis, causing irreversible damage to the tissues. The disease is rare, with an incidence of 10–40 patients per million per year. A recent meta-analysis has estimated the prevalence of the disease at 17.6 patients per 100 000, with 5 times more women affected than men.^[1]^ There have been improvements in patient survival over the years, with current estimates of 10 year survival being more than 70%.^[2]^ Mortality from this disease stems from organ involvement, with subsequent failure. SSc cardiac involvement represents an important contributor to overall mortality.

## Cardiac Complications Associated with Systemic Sclerosis

cardiac complications in SSc have been estimated to affect between 15%–30% of patients, though severity varies and are usually more common in patients with diffuse rather than limited cutaneous SSc.^[3]^ Autopsy studies have shown that between 60%–80% of SSc patients have some form of cardiac involvement such as myocarditis, pericarditis and myocardial fibrosis.^[4]^ Myocardial fibrosis has been noted on autopsy studies and with cardiac magnetic resonance imaging (cMRI) despite the absence of cardiac symptoms.^[4,5]^ Primary cardiac involvement in SSc is characterized by impaired microvascular perfusion, fibrosis and inflammation of the myocardium with histological analyses showing infiltration of immune cells, increased number of fibroblasts and formation of fibrous scars.^[6]^ Subsequently, these abnormal pathological processes manifest clinically as valvular dysfunction, ventricular dysfunction (more commonly diastolic over systolic dysfunction), pericardial effusion and electrical instabilities such as arrhythmias and conduction blocks.^[7]^ The degree of cardiac inflammation on histology correlated with the frequency of adverse cardiovascular outcomes, including heart failure, lethal arrhythmias needing implantable cardioverter defibrillators and deaths.^[6]^ Deaths from primary cardiac involvement usually occur from heart failure or lethal arrhythmias. In addition to primary cardiac involvement, patients with SSc are also at risk of coronary artery disease, with SSc patients having higher calcium scores compared to healthy controls.^[8]^ This review aims to summarize the evidence of markers that can be diagnostic (to confirm the presence of the condition), prognostic (to identify patients with higher risk of severe disease) or predictive (to determine response to treatment) of primary SSc cardiac involvement to guide future management.^[9]^

## B-type Natriuretic Peptide: Marker of Elevated Cardiac Wall Stress

B-type natriuretic peptide (BNP and N-terminal pro BNP (NT-proBNP) have been utilized as the typical diagnostic biomarkers for cardiac involvement in SSc. Cleavage of proBNP results in the biologically active 32 amino acid BNP and the biologically inactive 76 amino acid NT-ProBNP. Due to the longer half-life of NT-proBNP compared to BNP, levels of NT-proBNP are approximately six times higher in blood. BNP is released by cardiac myocytes in response to stretching caused by increased ventricular blood volume and serves to regulate blood pressure via salt and water homeostasis. BNP also has a role in cardiac remodeling, seen by how mice lacking the receptor for BNP develop cardiac hypertrophy and fibrosis.^[10]^ Both BNP and NT-ProBNP have been used clinically to evaluate for heart failure and left ventricular dysfunction outside of SSc.

NT-proBNP concentrations were higher in SSc patients than age- and sex-matched healthy controls.^[11,12]^ Higher levels of NT-proBNP were associated with abnormal echocardiographic findings, including valvular insufficiency and reduced left ventricular ejection fraction (LVEF).^[11]^ One study looking at 49 SSc patients without cardiac symptoms demonstrated that levels of BNP were increased in patients with late gadolinium enhancement (LGE). Levels of BNP also directly correlated with left ventricular mass index. which supports the utility of BNP as a diagnostic biomarker.^[5]^ Raised NT-ProBNP concentrations have also been shown to predict adverse cardiovascular outcomes and mortality.^[13,14]^ Higher rates of arrhythmia using both electrocardiogram and implantable loop recorders were found in SSc patients with elevated NT-proBNP, such as right bundle branch block and atrial arrhythmias.^[12,15]^ One study comparing 21 SSc patients with cardiac complications including arrhythmias, pericardial effusion or congestive heart failure compared with 42 SSc patients without cardiac symptoms showed significantly higher levels of NT-ProBNP in the group with cardiac complications.^[16]^ Patients with NT-ProBNP levels > 125 ng/L had higher risk of adverse events including myocarditis and arrhythmias, with 97.6% negative predictive value for mortality at 3 years. ^[14]^ This was supported in a multicentre prospective study of 523 SSc patients. Survivors at 3 years had significantly lower levels of NT-proBNP compared to patients who died. Taken together, this further highlights the utility of BNP and NT-proBNP as prognostic markers for SSc cardiac involvement. Levels of NT-ProBNP also fall following treatment, ^[17]^ which demonstrates its utility to monitor treatment response. Data on the correlation of BNP or NT-proBNP to severity on cardiac imaging has not been consistent,^[18]^ with further studies needed. Raised NT-proBNP is associated with severity of skin involvement and pulmonary hypertension,^[12]^ indicating its utility in SSc apart from primary cardiac involvement. Due to the significant utility of NT-ProBNP in predicting cardiac complications, the United Kingdom Systemic Sclerosis Study Group has recommended NT-proBNP to be checked at least annually.^[19]^

## Cardiac Troponins: Marker of Cardiac Injury

The combination of elevated BNP and troponins has been shown to be associated with worse survival rates.^[12]^ Troponins are muscle proteins that regulate the contractility of the cardiac muscles, and consist of three subunits: Troponins C, I and T. Troponin T (TnT) and Troponin I (TnI) have been extensively used in diagnosis of myocardial infarction, though more recent guidelines make use of high sensitivity cardiac troponin assays, which detects myocardial injury at much lower concentrations with the goal of making an earlier diagnosis.^[20]^ SSc studies have demonstrated similar findings with both TnI and TnT, although TnT is considered less specific for cardiac tissue as it is also released by skeletal muscle. A study of 272 patients investigating the relationship between TnI and SSc showed that patients with higher levels of TnI had higher prevalence of severe cardiomyopathy (defined as LVEF less than 35%) compared to the group with normal TnI levels.^[21]^ Furthermore, raised TnI was associated with worse cardiovascular outcomes, including increased risk of significant arrhythmias, higher right ventricular systolic pressure and increased risk of mortality.^[15,21]^ A retrospective cohort study demonstrated that levels of TnI were associated with the development of abnormal echocardiographic findings, with a proportion of these patients being initially asymptomatic. Levels of TnI subsequently decreased with treatment,^[22]^ which highlights the utility of TnI as a prognostic, diagnostic and response marker. Similar to TnI, TnT levels have been shown to be associated with lower LVEF, higher rates of arrhythmias and reduced long-term survival.^[12,23]^ One study evaluating 12 patients with SSc cardiac showed that TnT demonstrated greater correlation with cardiac involvement in SSc compared to NT-proBNP, and had superior diagnostic accuracy in cardiac dysfunction.^[24]^

Both NT-ProBNP and the cardiac troponins have thus been demonstrated to be associated with cardiac involvement in SSc, even when patients are asymptomatic and importantly, are prognostic, with higher levels indicative of poorer outcomes. These markers thus have great clinical utility in routine evaluation of SSc patients.

**Table 1:** Studies evaluating different biomarkers and cardiac involvement in systemic sclerosis.

Reference	Year	Study population	Biomarker	Finding
Sugiyama, *et al*.^[5]^	2019	49 SSc patients without cardiac symptoms	BNP	Higher levels of BNP in LGE+ group
Allanore, *et al*.^[14]^	2016	523 SSc patients	NT-proBNP	NT-ProBNP was associated with higher risk of mortality. No association between NT-ProBNP and LVEF or PAP
Chighizola, *et al*.^[16]^	2012	21 SSc patients with cardiac involvement vs 42 SSc patients without cardiac involvement	NT-ProBNP	Higher levels of NT-ProBNP in patients with cardiac involvement. Impairment in LVEF correlated with NT-ProBNP
Nordin, *et al*.^[11]^	2017	110 SSc patients	NT-proBNP, TnI	NT-ProBNP and TnI associated with valvular regurgitation, lower LVEF and higher PAP
Bissell, *et al*.^[15]^	2019	20 SSc patients without cardiac symptoms	NT-ProBNP, TnI	NT-ProBNP and TnI was associated with higher risk of significant arrythmia and LGE
Raluca, *et al*.^[13]^	2021	74 SSc patients without cardiac symptoms	NT-proBNP, TnI	NT-ProBNP and TnI associated with increased risk of cardiovascular events
He, *et al*.^[22]^	2023	21 SSc patients with cardiac involvement vs 63 SSc patients without cardiac involvement	NT-proBNP, TnI	Higher levels of NT-ProBNP and TnI in patients with cardiac involvement
Bosello, *et al*.^[12]^	2019	245 SSc patients	NT-proBNP, TnT	NT-ProBNP and TnT associated with lower LVEF, higher rate of RBBB and cardiac death
Barsotti, *et al*.^[24]^	2017	65 SSc patients	NT-ProBNP, TnT	Higher levels of NT-ProBNP and TnT in patients with cardiac involvement
Jha, *et al*.^[23]^	2022	675 SSc patients	NT-proBNP, TnT, CRP	NT-proBNP, TnT, CRP associated with higher risk of mortality. NT-proBNP and TnT associated with arrythmia. TnT was associated with systolic dysfunction
De Luca, *et al*.^[41]^	2022	19 SSc patients	NT-ProBNP, CRP	NT-ProBNP correlated with T1 mapping and ECV on cMRI. CRP correlated with T1 mapping
Paik, *et al*.^[21]^	2022	272 SSc patients	TnI	TnI associated with lower LVEF and higher RVSP
Abdel-Magied, *et al*.^[25]^	2016	20 SSc patients vs 10 healthy controls	IL-6	IL-6 correlated with mean and peak PAP
Jurisic, *et al*.^[26]^	2013	31 SSc patients vs 32 healthy controls	IL-6	IL-6 correlated with LVDD
Lin, *et al*.^[29]^	2019	105 SSc patients	IL-10	Higher IL-1β in patients with severe TR
Iannazzo, *et al*.^[31]^	2023	50 SSc patients vs 14 healthy controls	IL-33, sST2	Higher levels of sST2 in patients with LVDD. sST2 correlated with PAP. No association between LVDD and IL-33
Vertes, *et al*.^[34]^	2022	40 SSc patients	Galectin-3, sST2	Galectin-3 correlated with LVDD and grade of MR. No correlation between sST2 and echocardiography findings
Fukayama, *et al*.^[37]^	2020	40 SSc patients	IL-17F	IL-17F associated with elevated RVSP
Kosalka-Wegiel, *et al*.^[36]^	2024	43 SSc patients	IL-17	IL-17 associated with reduced LVEF and risk of mortality
Karadag, *et al*.^[39]^	2020	47 SSc patients vs 36 healthy controls	CRP, Galectin-3	CRP and Galectin-3 correlated with PSS
Jiang, *et al*.^[40]^	2022	95 SSc patients	CRP	CRP associated with reduced GLS
Rodriguez-Reyna, *et al*.[42]	2015	62 SSc patients	CRP	CRP associated with myocardial fibrosis on cMRI
Gieszczyk, *et al*.^[49]^	2013	49 SSc patients vs 20 healthy controls	GDF15, TGFβ1	GDF15 correlated with LVDD No association between TGFβ1 and echocardiography findings
Hromádka, *et al* .[48]	2017	33 SSc patients vs 20 healthy controls	GDF15, Galectin-3	GDF15 and Galectin-3 correlated with ECV and native T1 on CMRI and GLS on echocardiography.
Tennøe, *et al* .[50]	2022	371 SSc patients	ANGPT2, OPN, and TRAIL	ANGPT2, OPN, and TRAIL associated with LVDD and RV systolic dysfunction. ANGPT2 and OPN associated with GLS
El-Adili, *et al*.^[54]^	2022	106 SSc patients vs 22 healthy controls	Periostin	Periostin correlated with LV mass and LV mass index

ANGPT2, Angiopoietin 2; cMRI, cardiac magnetic resonance imaging; CRP, C-reactive protein; ECV, extracellular volume; GDF15, growth differentiation factor 15; GLS, global longitudinal strain; LGE: late gadolinium enhancement; LV, left ventricle; LVDD, left ventricular diastolic dysfunction; LVEF, left ventricular ejection fraction; MR, mitral regurgitation; NT-proBNP, N-terminal pro B-type natriuretic peptide; OPN, osteopontin; PAP, pulmonary artery pressure; PSS, peak systolic strain; RVSP, right ventricular systolic pressure; SSc: systemic sclerosis; TGFβ1: transforming growth factor β1; TR, tricuspid regurgitation; TRAIL, tumor necrosis factor-related apoptosis-inducing ligand; RV, right ventricle; TnI, troponin-I; TnT, troponin-T.

## Pro-inflammatory Cytokines: Marker of Inflammation

Pro-inflammatory cytokines play a key role in the pathogenesis of SSc. In this next section, we will highlight certain cytokines that may be implicated in SSc cardiac involvement, with potential role as biomarkers. Interleukin (IL)-6 is a proinflammatory cytokine that is produced by many different cells including endothelial cells, dendritic cells, macrophages and lymphocytes in response to tissue injury or infection. In the heart, IL-6 stimulates leukocyte recruitment and proliferation, and induces collagen production resulting in downstream cardiac remodeling. Serum IL-6 levels were higher in SSc patients compared to healthy controls, and correlated with high resolution computed tomography (HRCT) scores and peak pulmonary artery pressures.^[25]^ One study evaluated 31 SSc patients with preserved LVEF, and demonstrated that IL-6 levels correlated with the severity of left ventricular diastolic dysfunction on echocardiography.^[26]^ Tocilizumab, an antibody against IL-6 receptor, has been used to treat SSc with good outcomes particularly with respect to lung involvement.^[27]^ Two case reports focusing on the role of tocilizumab in the treatment of SSc cardiac complications demonstrated improvement in NT-proBNP and troponin levels, left and right ventricular ejection fractions and imaging parameters on cMRI following treatment (improvement in native T1 and stabilization/improvement in extracellular volume fraction).^[17,28]^ Taken together, IL-6 may be a predictive and prognostic biomarker, and also represents a novel therapeutic target for SSc cardiac involvement. Further studies would be beneficial to elucidate if IL-6 could also be a diagnostic marker for cardiac involvement in SSc.

Two other groups of cytokines that have been evaluated in SSc are IL-1 and IL-1). IL-1 exists as a large family of ligands. Two of the well characterized members of this family, IL-1α and IL-1β, have been shown to promote inflammation and fibrosis in SSc, including the heart.^[29]^ Levels of various other IL-1 family cytokines, such as IL-18 and IL-33, have been shown to be increased in both sera and tissue samples from the heart, skin and lung.^[30]^ Raised levels of both IL-1β and IL-33 have been associated with cardiac dysfunction in SSc patients, particularly valvular heart disease and diastolic dysfunction.^[29,31]^ IL-33 binds to suppression of tumorigenicity 2 (ST2) receptor. ST2 exists as a transmembrane form and a soluble form, soluble ST2 (sST2). sST2 binds to IL-33 as a decoy receptor, interfering with normal IL-33/ST2 signaling. sST2 levels have been associated with increased microvascular injury as seen on nailfold microscopy.^[32]^ sST2 levels correlated with the degree of myocardial fibrosis and inflammation evidenced by cMRI findings of increased extracellular volume, oedema and late gadolinium enhancement. ^[33]^ A study of 50 SSc patients demonstrated increased sST2 levels in patients with diastolic dysfunction compared to patients without.^[31]^ However, another study did not find any association between sST2 levels and left ventricular dysfunction on echocardiography, possibly due to the reduced sensitivity of echocardiography in detecting early SSc cardiac involvement.^[34]^

IL-17 is released by Th17 cells, and is also increased in sera and skin samples of SSc patients.^[35]^ IL-17 acts as a downstream effector of IL-1 signaling and mediates increased collagen expression, thus promoting the profibrotic process in SSc. Higher levels of IL-17 have been associated with reduced LVEF and increased right ventricle systolic pressures on echocardiography,^[36,37]^ and correlated with severity of interstitial lung disease and skin scores. There are currently ongoing studies with IL-17 blockade using brodalumab in SSc, for which preliminary Phase I data has demonstrated improvement in skin scores. ^[38]^ It would be of interest to further study IL-17 as both a biomarker and a therapeutic target for SSc and its cardiac complications.

C-reactive protein (CRP), is an acute phase reactant that is released by the liver in response to increased proinflammatory cytokines, particularly IL-1 and IL-6. Studies have shown a correlation between CRP and ventricular impairment measured through echocardiographic longitudinal strain, which is a more sensitive marker of left ventricular dysfunction before overt reduction in LVEF.^[39,40]^ Studies looking at myocardial inflammation and fibrosis using cMRI have similarly demonstrated a correlation with CRP levels.^[41,42]^ Higher levels of CRP are associated with reduced 5-year survival in SSc patients, particularly with interstitial lung disease (ILD).^[43]^ Two studies demonstrated that persistent CRP of greater than 5 mg/L was a poor prognostic marker, being associated with more difficult to treat disease, higher skin scores, faster declines in forced vital capacity and overall mortality.^[44,45]^

## Profibrotic Cytokines: Marker of Tissue Injury

Profibrotic cytokines such transforming growth factor β (TGFβ) are key drivers of the disease process in SSc. Downstream signaling of TGF-β results in activation of myofibroblasts and excessive production of extracellular matrix proteins which drive fibrosis. Patients with diffuse cutaneous SSc and more significant lung involvement had higher levels of TGF-β compared to those with limited cutaneous SSc and healthy controls. However, no relationship could be identified between TGF-β levels and cardiac features in SSc patients compared to healthy controls.^[46]^ Growth/differentiation factor 15 (GDF-15), a protein that is part of the TGF-β superfamily, has been identified as a predictor of hospitalization and mortality due to heart failure outside of SSc. Levels of GDF-15 are increased in the sera of SSc patients and are associated with worse outcomes in interstitial lung disease and pulmonary arterial hypertension.^[47]^ GDF-15 positively correlated with extracellular volume and native T1 mapping on cMRI^[48]^ and left ventricular dysfunction on echocardiography.^[49]^

## Novel Markers of Cardiac Involvement in Systemic Sclerosis

More recently, separate SSc cohort studies have identified new biomarkers to assess SSc cardiac involvement. These include osteopontin and angiopoietin 2, levels of which were associated with biventricular dysfunction.^[50]^ Apart from cardiac dysfunction, osteopontin was demonstrated to be raised in the plasma and skin of SSc patients compared to healthy controls and was associated with pulmonary fibrosis.^[51,52]^ The same study identified tumor necrosis factor-related apoptosisinducing ligand (TRAIL) to be associated with right ventricular dysfunction and separately shown to correlate with severity of pulmonary hypertension.^[53]^ Periostin is another new biomarker that was shown to be increased in SSc cardiac tissue and correlated with left ventricular dysfunction.^[54]^ Galectin-3 has been identified in two separate SSc cohorts to correlate with cardiac dysfunction, being associated with both echocardiographic and CMRI abnormalities.^[34,48]^ Additional studies would be needed to further characterize these markers.

Platelet derived growth factor (PDGF) and Vascular Endothelial Growth Factor (VEGF) are growth factors that have been implicated in SSc through promoting fibrosis. However, no study has directly investigated levels of PDGF and VEGF with cardiac disease in SSc patients. PDGF regulates fibroblasts and mesenchymal stem cells, with a role in cardiac remodeling such as after myocardial infarction. Levels of PDGF and its receptors have been shown to be elevated in SSc patients, both in tissue samples and in blood.^[55]^ A preclinical study evaluating crenolanib, a tyrosine kinase inhibitor against the PDGF receptor, showed improvements in skin and heart fibrosis in mice treated with crenolanib versus control.^[56]^ VEGF is an angiogenic growth factor, with elevated levels in blood and skin samples from SSc patients compared to healthy controls ^[57]^ Levels of VEGF have been shown to correlate with severity of pulmonary hypertension.^[58]^ In vitro, VEGF drives fibrosis in human atrial fibroblasts and overexpression of VEGF in mice promotes cardiac fibrosis.^[59,60]^ These factors thus represent potential biomarkers that can be evaluated in future.

## Conclusion

In conclusion, several biomarkers for cardiac involvement in SSc are available and may also be useful in the assessment of other systemic involvement such as skin and lung. Biomarkers that are recommended for routine care include BNP/NT-proBNP and cardiac troponins. Biomarkers with high potential for translation such as IL-6 and IL-17, have direct roles in disease pathogenesis and thus also represent therapeutic targets. Further studies will be needed to assess if they could represent predictive biomarkers with treatment. Other markers remain in the investigational stage. Well-designed studies to validate cardiac biomarkers for purposes of diagnosis, prognosis and prediction of treatment response are needed to advance the management of SSc cardiac involvement.
